# NOP56 Drives Colorectal Cancer Progression by Modulating p53 Acetylation through SIRT1/p300

**DOI:** 10.7150/ijbs.126463

**Published:** 2026-04-16

**Authors:** Ji Eon Park, Chi-Hoon Ahn, Deok Yong Sim, Su-Yeon Park, Hyun Ju Cha, Bum-Sang Shim, Bonglee Kim, Sung-Hoon Kim

**Affiliations:** 1 Cancer Molecular Targeted Herbal Research Laboratory, College of Korean Medicine, Kyung Hee University, 1 Hoegi-dong, Dongdaemun-gu, Seoul 02447, South Korea.; 2 South Baylo University, Suite 500, 4055 Wilshire Blvd, Los Angeles, CA90010, USA.

**Keywords:** NOP56, colorectal cancer, apoptosis, p53 acetylation, SIRT1, p300

## Abstract

Nucleolar protein 56 (NOP56), a core component of small nucleolar ribonucleoprotein complexes, has been implicated in oncogenesis through the regulation of reactive oxygen species (ROS) homeostasis; however, its role in colorectal cancer (CRC) remains unclear. Here, we investigated the clinical relevance and biological function of NOP56 in CRC using TCGA datasets, tissue microarrays, next-generation sequencing, and *in vitro* and *in vivo* models. NOP56 expression was markedly elevated in CRC tissues compared with adjacent normal tissues and was correlated with poor patient survival. Silencing of NOP56 suppressed cell viability, colony formation, and migration, particularly in p53 wild-type HCT116 cells, and altered gene expression programs related to DNA damage response and apoptosis. Mechanistically, NOP56 depletion induced cell cycle arrest and apoptosis, accompanied by increased p53 and p21 levels and reduced expression of pro-caspase-3, c-Myc, Cyclin E, CDK2, CDK4, MDM2, and SIRT1. Conversely, NOP56 overexpression promoted p53 degradation, whereas its knockdown enhanced p53 stability and acetylation through suppression of SIRT1 and activation of p300, as supported by evidence of direct interaction and colocalization. Furthermore, NOP56 silencing synergistically enhanced the cytotoxic effect of 5-fluorouracil (5-FU). In a xenograft model, NOP56 knockdown markedly reduced tumor growth as well as PCNA and SIRT1 expression, while increasing p53 and cleaved caspase-3 levels. Collectively, these findings identify NOP56 as an oncogenic driver that promotes CRC progression by inducing p53 degradation, whereas its inhibition triggers apoptosis via p53 acetylation regulated by the SIRT1/p300 axis, highlighting NOP56 as a promising therapeutic target for p53 wild-type CRC.

## Introduction

Colorectal cancer (CRC) is the third most common malignancy in the United States, with an estimated 153,020 new cases in 2023 1, 2. Alarmingly, approximately 11% of cases occur in patients younger than 50 years, with incidence rates in this population increasing by 1-2% annually 3. Current therapies include 5-fluorouracil (5-FU) and targeted agents such as RAF inhibitors (encorafenib), VEGF inhibitors (bevacizumab), and EGFR inhibitors (cetuximab) 4-6. In addition, a variety of pro-apoptotic agents have been developed to induce cancer cell death through caspase-dependent or caspase-independent mechanisms, often involving activation of a tumor suppressor such as p53 7, 8 or inhibition of oncogenic pathways, including c-Myc and the CCR4-NOT transcription complex subunit 2 (CNOT2) 9-11.

The tumor suppressor p53 is a transcription factor that regulates diverse cellular processes, including cell cycle progression, apoptosis, autophagy, DNA repair, and metabolism 12-14. Under physiological conditions, p53 protein levels are tightly controlled by MDM2 and MDMX through ubiquitin-mediated degradation 15, 16. In response to intracellular or extracellular stress, however, p53 is rapidly stabilized and activated through multiple post-translational modifications 17, 18. Among these, acetylation at specific lysine residues (K120, K164, K370, K372, K373, K381, and K382) enhances p53 transcriptional activity and is mediated by acetyltransferases such as p300/CREB-binding protein (CBP)/PCAF and Tip60/MOF/MOZ 19, 20. Conversely, silent information regulator 1 (SIRT1) deacetylates p53, thereby suppressing its tumor-suppressive function and promoting oncogenesis 21, 22.

Nucleolar protein 56 (NOP56), also known as NOL5A or SCA36, is a core component of the small nucleolar ribonucleoprotein complex (snoRNP) involved in pre-rRNA processing 23. Aberrant overexpression of NOP56 has been reported in Burkitt's lymphoma 24, cervical cancer 25, and hepatocellular carcinoma 26. Furthermore, NOP56 downregulation was shown to induce apoptosis through reactive oxygen species (ROS) accumulation in KRAS-mutant lung cancer 21, while Mahajan *et al.* identified NOP56 as a potential diagnostic and prognostic biomarker in colorectal cancer 27. Nonetheless, the mechanistic role of NOP56 in CRC remains poorly defined.

Therefore, in the present study, we investigated the oncogenic function of NOP56 in CRC, focusing on its regulation of p53 acetylation via p300 and SIRT1. Using both *in vitro* and *in vivo* models, we elucidated a novel NOP56-mediated mechanism underlying CRC progression.

## Materials and Methods

### Cell lines and culture

Human colorectal cancer cell lines HCT116, SW480, SW620, and HT-29 were obtained from the Korean Cell Line Bank (KCLB, Seoul, Korea). Cells were cultured in RPMI-1640 medium (Welgene, Gyeongsan, Korea) supplemented with 10% fetal bovine serum (FBS) and 1% antibiotic-antimycotic solution (penicillin, streptomycin, amphotericin B). Human colon fibroblast CCD-18Co cells were maintained in Dulbecco's Modified Eagle's Medium (DMEM) with 10% FBS and 1% antibiotic-antimycotic solution. All cells were incubated at 37 °C in a humidified 5% CO₂ atmosphere.

### RNA interference and plasmid transfection

Cells were seeded overnight and transfected with NOP56 siRNA, MDM2 siRNA, p300 siRNA, SIRT1 siRNA or negative control siRNA (Bioneer, Daejeon, Korea) using INTERFERin® (Polyplus, France) according to the manufacturer's protocol. Expression vectors for NOP56, HA-ubiquitin, Flag-p53 and SIRT1, as well as the empty vector pcDNA3.0, were obtained from Addgene (MA, USA).

### TCGA data analysis and survival analysis

NOP56 mRNA expression in CRC was analyzed using datasets from The Cancer Genome Atlas (TCGA). Survival outcomes, including disease-free survival (DFS) and overall survival (OS), were assessed by Kaplan-Meier analysis using RStudio software.

### Tissue microarray and immunohistochemistry

As previously described 28, 29, human colon tissue microarray (Colon tumor test tissue microarray, T053b) was purchased from tissuearray.com. Tissue sections (5 µm) were deparaffinized, rehydrated, and immunostained with an anti-NOP56 antibody (1:500) at 4 °C overnight, followed by incubation with a secondary antibody at room temperature for 1 h. Immunoreactivity was visualized using DAB, and stained sections were scanned using a Leica SCN400 slide scanner.

### RNA sequencing and data analysis

According to NGS analysis 30, total RNA was isolated from HCT116 cells transfected with negative control or NOP56 siRNA using TRIzol reagent. RNA integrity was assessed using an RNA 6000 Nano Chip on the Bioanalyzer system (Agilent). Libraries were prepared using the QuantSeq 3′ mRNA-Seq kit (Lexogen, Vienna, Austria) and sequenced by e-Biogen (Seoul, Korea). Differentially expressed genes were analyzed for functional annotation using the DAVID and Medline databases.

### Cell viability assay

As previously described 31, HCT116 and SW620 cells (1×10⁴ cells/well) were seeded into 96-well plates and incubated for 24-72 h. MTT (1 mg/mL, 20 µL) was added for 2 h, and formazan crystals were dissolved in DMSO (100 µL). Absorbance was measured at 570 nm (Bio-Rad, CA, USA).

### Colony formation assay

As previously described 32, HCT116 cells (1×10³ cells/well) were seeded in 6-well plates and cultured for 14 days. Colonies were fixed and stained with Diff-Quick solution (Sysmex, Kobe, Japan) and subsequently counted.

### Wound healing assay

As previously described 33, scratches were made using pipette tips in confluent monolayers of HCT116 cells transfected with negative control or NOP56 siRNA. At 72 h after scratching, cell migration into the wound area was quantified.

### Cell cycle analysis

As previously described 34, transfected HCT116 cells were fixed in 70% ethanol overnight at -20 °C, treated with RNase A (100 µg/mL) at 37 °C for 50 min, and stained with propidium iodide (50 µg/mL). DNA content was analyzed by flow cytometry using a BD Biosciences FACSCalibur system.

### Western blotting

As previously described 35, 36, cell lysates were prepared in RIPA buffer supplemented with protease and phosphatase inhibitors. Proteins were separated by SDS-PAGE, transferred to nitrocellulose membranes, and probed with primary antibodies against NOP56 (Cat. No. NBP1-46847, Novus Biologicals, USA; and Cat. No. 18181-1-AP, Proteintech, USA), PARP (Cat. No. 9542), Caspase-3 (Cat. No. 9662), CDK2 (Cat. No. 2546), CDK4 (Cat. No. 12790), MDM2 (Cat. No. 86934), GST-Tag (Cat. No. 2625) and Acetyl-p53 (K382) (Cat. No. 2525) from Cell Signaling Technology (USA); Bcl-2 (Cat. No. sc-492), p53 (Cat. No. sc-126), p27 (Cat. No. sc-527), Cyclin E (Cat. No. sc-481), HA (Cat. No. sc-7392), and Flag (Cat. No. sc-807) from Santa Cruz Biotechnology (USA); Ubiquitin (polyclonal) (Cat. No. PA1-187) from Invitrogen (USA); c-Myc (Cat. No. ab32072) from Abcam (USA); SIRT1 (Cat. No. 13161-1-AP) from Proteintech; p300 (Cat. No. 05-257) from Millipore (USA); and β-actin (Cat. No. A1978) from Sigma-Aldrich (USA). Detection was performed using HRP-conjugated secondary antibodies and enhanced chemiluminescence, and signals were captured using an Amersham Imager 600 (Cytiva, Germany).

### Real-time quantitative PCR (qRT-PCR)

As previously described 37, total RNA was extracted using QIAzol reagent (Qiagen, USA) and reverse-transcribed using M-MLV reverse transcriptase. Quantitative real-time PCR was performed using a LightCycler™ system (Roche, Basel, Switzerland) with gene-specific primers purchased from Bioneer (Daejeon, Korea). The primer sequences were as follows:

NOP56 forward, 5′-GGCTAAGGCTATTCTGGATGCC-3′; NOP56 reverse, 5′-TGTGTAGGCTCTGGCGGTATTC-3′; TP53 forward, 5′-CCTCAGCATCTTATCCGAGTGG-3′; TP53 reverse, 5′-TGGATGGTGGTACAGTCAGAGC-3′; MYC forward, 5′-CCTGGTGCTCCATGAGGAGAC-3′; MYC reverse, 5′-CAGACTCTGACCTTTTGCCAGG-3′.

### Cycloheximide chase assay

As previously described 38, HCT116 cells were transfected with siNC or siNOP56 for 48 h and then treated with 50 µg/mL cycloheximide (Merck, Darmstadt, Germany) for the indicated time points. Protein stability was assessed by Western blotting.

### Computational docking study

Crystal structures of p53 (DNA-binding domain; PDB: 1TUP) and NOP56 (N-terminal domain; PDB: 6ZDT) were retrieved from the RCSB Protein Data Bank and visualized in PyMOL (Schrödinger, LLC). Structures were preprocessed by removing water molecules, adding polar hydrogens, and assigning Gasteiger charges. The two proteins were then structurally aligned using PyMOL to assess potential complementary surfaces.

### Co-immunoprecipitation (Co-IP)

As previously described 39, 40, cell lysates (250 µg) were incubated with antibodies against NOP56, MDM2, p53, HA, or Flag, followed by incubation with protein A/G agarose beads (Santa Cruz Biotechnology, USA) at 4 °C. Immune complexes were washed and analyzed by Western blotting.

### Immunofluorescence

As previously described 41, transfected HCT116 cells were fixed with 4% formaldehyde, permeabilized, and incubated with primary antibodies against **NOP56 and p53**, followed by incubation with Alexa Fluor-conjugated secondary antibodies and DAPI. Images were acquired using an FV10i confocal microscope (Olympus, Tokyo, Japan).

### GST pull-down assay

GST-fusion proteins containing individual domains of p53 were generated and obtained from Bioneer (Daejeon, Korea). Each GST-p53 domain fusion protein was expressed in *Escherichia coli* BL21 cells and purified using Pierce™ Glutathione Magnetic Agarose Beads (Thermo Fisher Scientific) according to the manufacturer's instructions. Bead-bound GST-p53 domain fusion proteins were incubated with HCT116 cell lysates at 4 °C for 4 h with gentle rotation. After extensive washing, bound proteins were eluted by boiling in SDS sample buffer and analyzed by SDS-PAGE followed by western blotting.

### Ubiquitination assay

SW480 or HCT116 cells were transfected with siNC, siNOP56, or OE-NOP56, together with HA-ubiquitin. Cells were treated with 20 μM MG132 for 2 h prior to harvest, lysed, and subjected to immunoprecipitation using an anti-HA antibody and protein G-agarose beads. Immunoprecipitates were analyzed by Western blotting using an anti-HA antibody.

### Xenograft tumor model

Animal experiments were approved by the Kyung Hee University Institutional Animal Care and Use Committee (IACUC; approval number KHUASP(SE)23-257). HCT116 cells (4×10⁶) transfected with scrambled shRNA or NOP56 shRNA were subcutaneously injected into male BALB/c nude mice. Tumor volumes were calculated using the formula (length × width²)/2. After 35 days, tumors were excised, weighed, and analyzed by immunohistochemistry for NOP56, p53, SIRT1, PCNA, and cleaved caspase-3.

### Statistical analysis

All experiments were performed with at least three independent replicates. Data are expressed as means ± standard deviation (SD). Statistical analyses were performed using GraphPad Prism version 8 (GraphPad Software, San Diego, CA, USA). Differences among multiple groups were evaluated using one-way analysis of variance (ANOVA) followed by Tukey's post hoc test. Comparisons between two groups were performed using Student's t-test. A value of p < 0.05 was considered statistically significant. TCGA survival analyses were performed using the Kaplan-Meier method with R software (R Foundation for Statistical Computing).

## Results

### NOP56 is overexpressed in human colorectal cancer tissues and cell lines and correlates with poor survival

**Figure 1:**
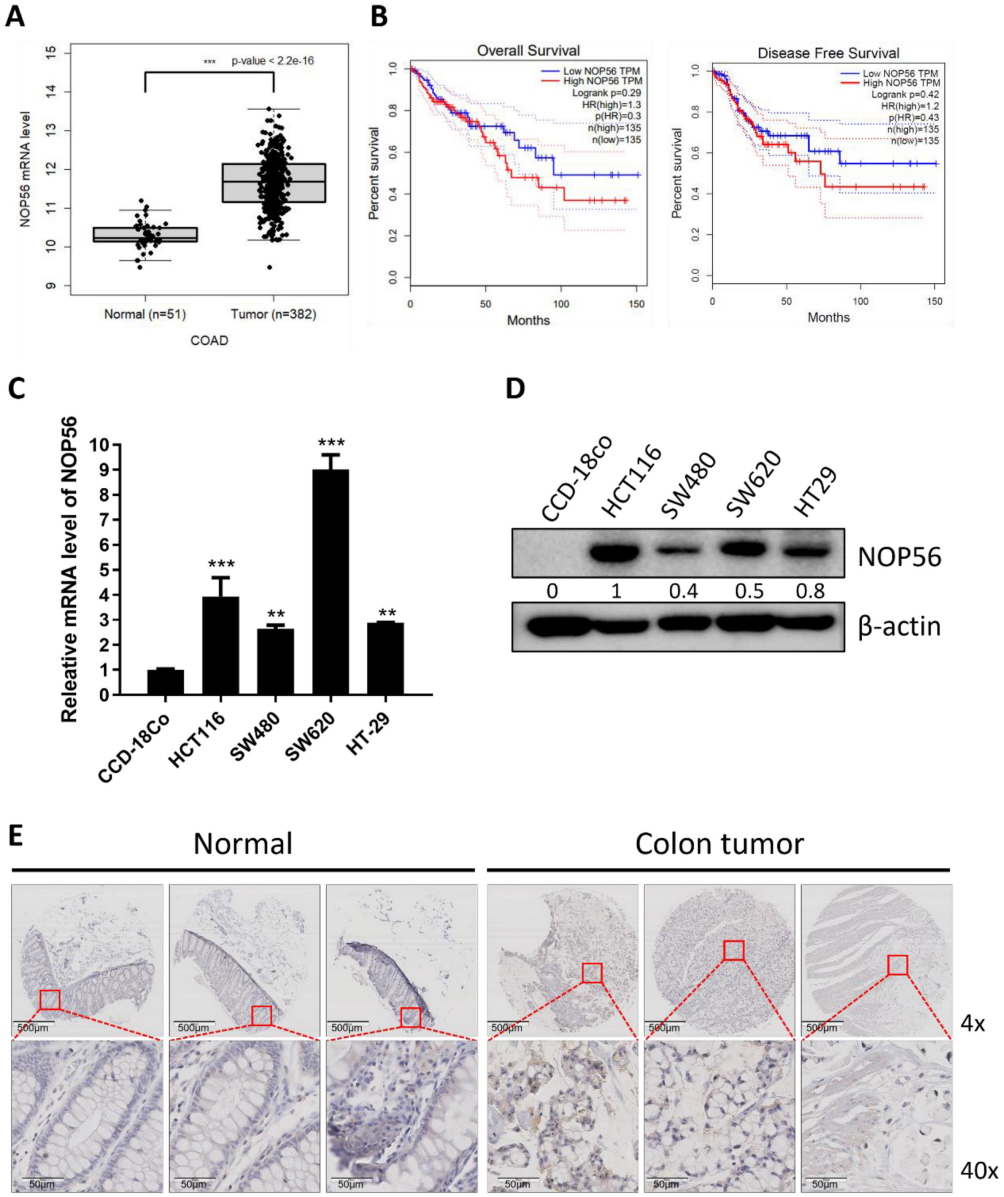
** NOP56 is overexpressed in colorectal cancer cell lines and tissues. (A)** The expression of NOP56 is upregulated at mRNA level in colorectal cancer tissues compared to adjacent normal colon tissues by TCGA database. **(B)** The higher expression level of NOP56 predicts the poor prognosis of DFS and OS in CRC patients. **(C)** NOP56 mRNA level in colorectal cancer cell lines by qRT-PCR. **(D)** Endogenous expression levels of NOP56 in colorectal cancer cell lines by Western blotting. **(E)** Representative immunohistochemical images showing NOP56 expression in human colon tumor tissues and adjacent normal colon tissues from a tissue microarray. Data are expressed as means ± SD. **p < 0.01, ***p < 0.001 vs normal CCD-18Co cells. All experiments were repeated three times independently.

Analysis of TCGA datasets revealed that NOP56 mRNA expression was significantly higher in CRC tissues compared with adjacent normal colon tissues (Figure 1A). Kaplan-Meier survival analysis further demonstrated that both OS and DFS were markedly reduced in patients with high NOP56 expression compared with those with low expression (Figure 1B), which was not intended for multivariable prognostic modeling.

Consistently, endogenous NOP56 expression at both the mRNA and protein levels was elevated in colorectal cancer cell lines (HCT116, SW480, SW620, and HT-29) compared with normal human colon fibroblast CCD-18Co cells (Figure 1C, D). Among these cell lines, NOP56 expression in SW480 cells was detectable but markedly lower than in HCT116, SW620, and HT-29, limiting their suitability for functional studies. Although HT-29 cells exhibited relatively high NOP56 expression, they harbor mutant p53, making them less appropriate for analyses focused on p53 acetylation and ubiquitination. Therefore, HCT116 (p53 wild-type) was selected as the primary model, while SW620 (p53 mutant) was included as a comparative model to assess p53 dependency. HCT116 and SW620 cells exhibited relatively higher levels of NOP56 expression. Supporting these findings, tissue microarray analysis further demonstrated stronger NOP56 expression in CRC tissues compared with adjacent normal colon tissues (Figure 1E).

### NOP56 depletion inhibits proliferation and induces apoptosis in HCT116 and SW620 cells

**Figure 2:**
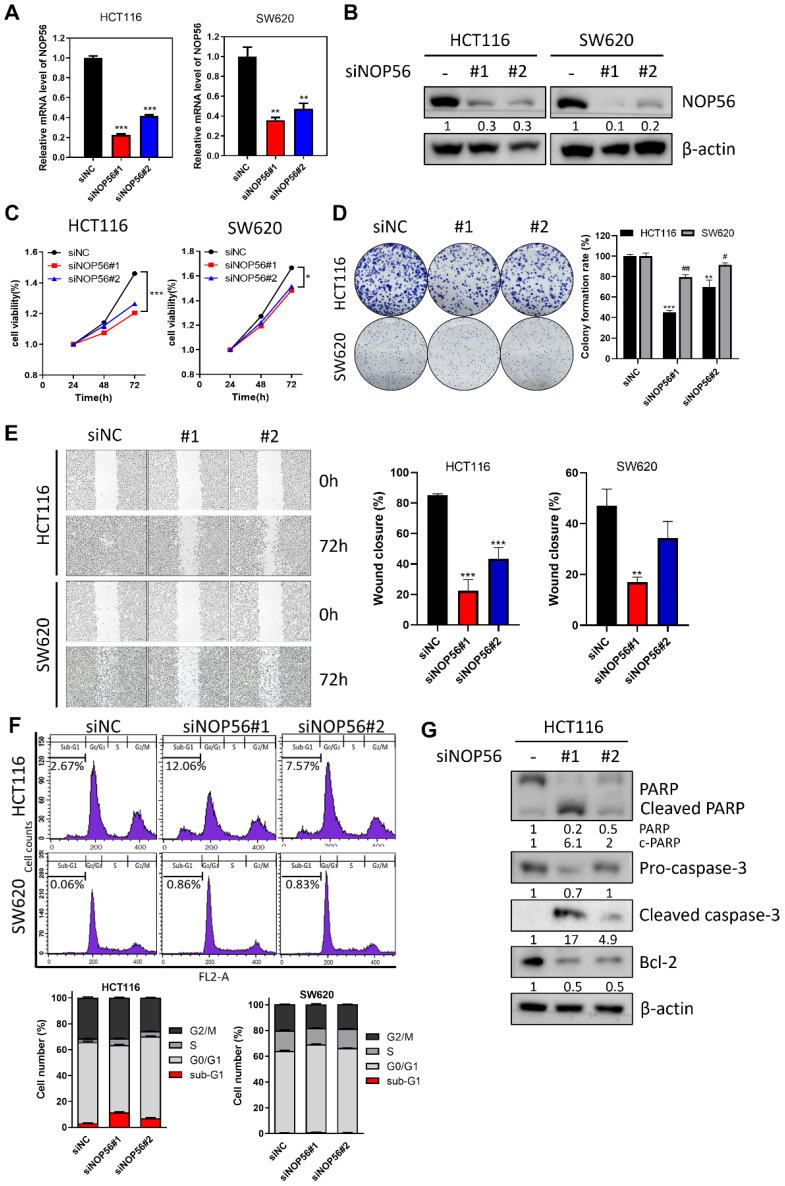
** Effects of NOP56 depletion on proliferation, migration, apoptosis, and sub-G1 population in colorectal cancer cells. (A, B)** HCT116 and SW620 cells were transfected with negative control or NOP56 siRNA. NOP56 expression at the mRNA (A) and protein (B) levels was analyzed by RT-qPCR and Western blotting. **(C)** Effect of NOP56 depletion on the viability of HCT116 and SW620 cells, as determined by MTT assay over the indicated time course at 37 °C. **(D)** Effect of NOP56 depletion on colony formation in HCT116 and SW620 cells, as assessed by crystal violet staining. **(E)** Effect of NOP56 depletion on the migratory activity of HCT116 and SW620 cells, as evaluated by wound healing assay. **(F)** Effect of NOP56 depletion on the sub-G1 population in HCT116 and SW620 cells, as analyzed by flow cytometry. **(G)** Effect of NOP56 depletion on apoptosis-related proteins in HCT116 cells. Cell lysates were subjected to Western blotting for PARP (poly(ADP-ribose) polymerase), Caspase-3 (cysteine-aspartic protease-3), Bcl-2 (B-cell lymphoma 2), and β-actin as a loading control. Data are presented as mean ± SD. **p < 0.01, ***p < 0.001 vs. untreated control; #p < 0.05, ##p < 0.01 vs. untreated control.

To investigate the functional role of NOP56 in colorectal cancer, HCT116 (p53 wild-type) and SW620 (p53 mutant) cells were transfected with NOP56 siRNA. Transfection efficiency was confirmed by RT-qPCR and Western blotting, demonstrating effective depletion of NOP56 at both the mRNA and protein levels (Figure 2A, B).

NOP56 knockdown significantly reduced cell proliferation in a time-dependent manner, with a stronger inhibitory effect observed in HCT116 cells compared with SW620 cells (Figure 2C). Consistently, colony formation was markedly reduced following NOP56 silencing, with a greater reduction in HCT116 cells compared with SW620 cells (Figure 2D). Wound healing assays further demonstrated that NOP56 depletion significantly suppressed migratory activity in both cell lines compared with siNC (Figure 2E).

To determine whether NOP56 depletion induces apoptosis, cell cycle distribution and apoptosis-related markers were analyzed. In HCT116 cells, NOP56 knockdown increased the proportion of sub-G1 phase cells to 12.06% and 7.57%, compared with 2.67% in siNC. In contrast, SW620 cells displayed only a modest increase in sub-G1 phase (0.86% and 0.83% vs. 0.06% in controls) (Figure 2F). Western blot analysis further confirmed apoptotic induction, as evidenced by reduced levels of PARP, pro-caspase-3, and Bcl-2, along with increased expression of cleaved PARP and cleaved caspase-3 (Figure 2G).

These findings indicate that NOP56 depletion suppresses proliferation, clonogenicity, and migration while inducing apoptosis, with more pronounced effects observed in p53 wild-type HCT116 cells compared with p53-mutant SW620 cells.

### Differentially expressed gene profiles in NOP56-depleted HCT116 cells

**Figure 3:**
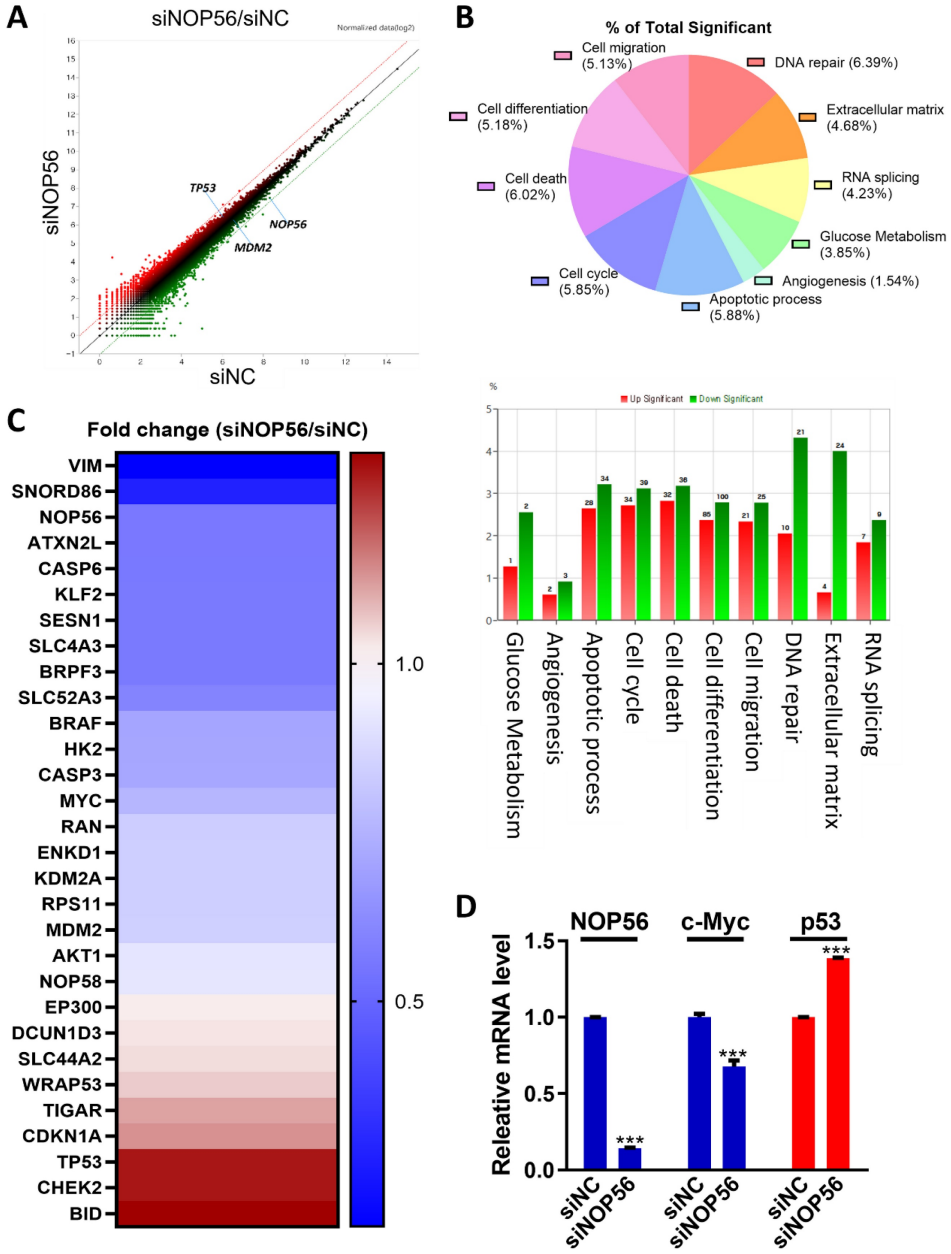
** Differentially expressed gene profiles in NOP56-depleted HCT116 cells. (A)** Scatter plot showing fold changes in gene expression in NOP56-depleted HCT116 cells compared with negative control-transfected cells. **(B)** Gene ontology (GO) analysis of significantly enriched signaling pathways in HCT116 cells following NOP56 depletion. Upregulated pathways are shown in red, and downregulated pathways are shown in green. **(C)** Heatmap of differentially expressed genes in NOP56-depleted HCT116 cells. Upregulated genes are shown in red, and downregulated genes are shown in blue. **(D)** Effect of NOP56 depletion on c-Myc and p53 mRNA expression in HCT116 cells, as determined by qRT-PCR analysis. Data are presented as mean ± SD. ***p < 0.001 vs. negative control.

To identify NOP56-associated genes and signaling pathways, next-generation sequencing (NGS) analysis was performed in HCT116 cells transfected with NOP56 siRNA. Efficient knockdown of NOP56 was confirmed at the mRNA level. Scatter plot analysis revealed a marked decrease in NOP56 and MDM2 expression, accompanied by more than a twofold increase in p53 expression (Figure 3A).

Gene ontology (GO) analysis indicated significant enrichment of pathways related to DNA damage (6.39%), cell death (6.02%), apoptosis (5.88%), and extracellular matrix regulation (4.68%) (Figure 3B). mRNA sequencing further demonstrated distinct expression profiles of upregulated (red) and downregulated (blue) genes in NOP56-depleted cells (Figure 3C).

Validation by qRT-PCR confirmed upregulation of p53 and downregulation of NOP56 and c-Myc in NOP56-silenced HCT116 cells, consistent with the NGS results (Figure 3D).

### NOP56 regulates p53 and its downstream targets in HCT116 cells

**Figure 4:**
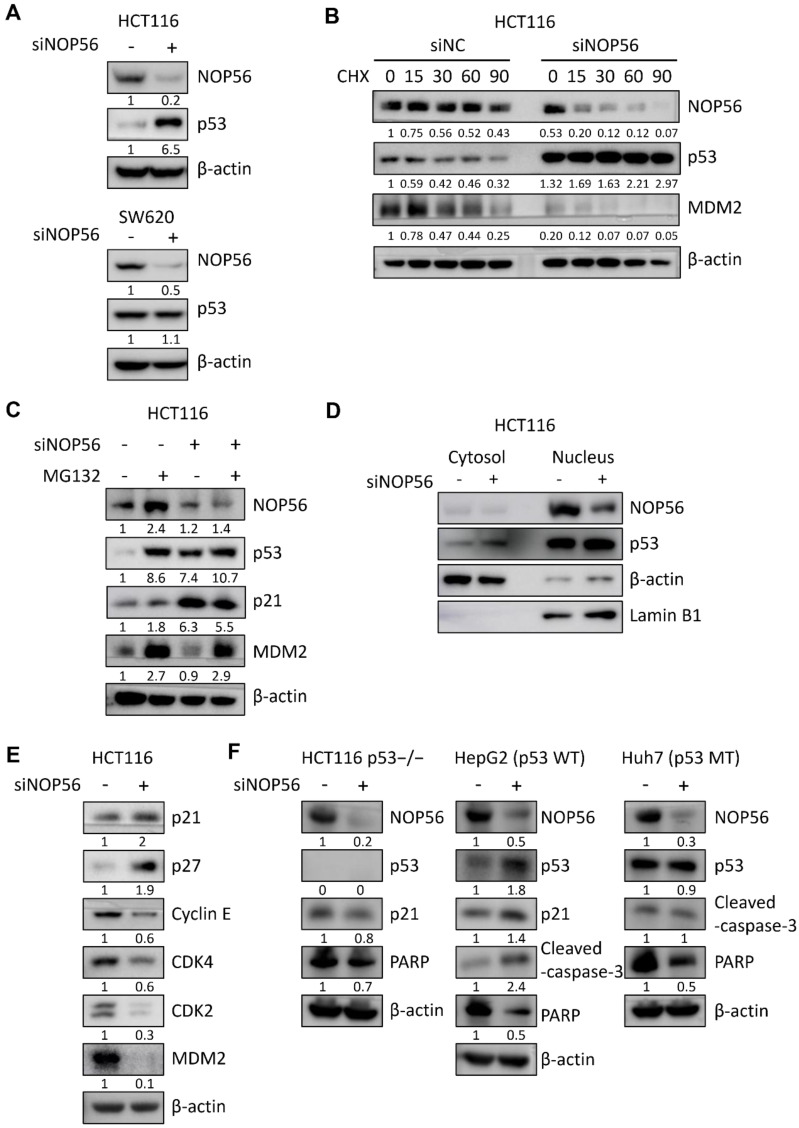
** NOP56 depletion activates p53 signaling and stabilizes p53 in HCT116 cells. (A)** Effect of NOP56 depletion on p53 protein expression in HCT116 and SW620 cells. **(B)** Effect of NOP56 depletion on the stability of p53 and MDM2 in HCT116 cells treated with cycloheximide (CHX). Cells were transfected with negative control or NOP56 siRNA and treated with CHX (50 μg/mL) for the indicated time points (0, 15, 30, 60, and 90 min), followed by Western blot analysis. **(C)** Effect of the proteasome inhibitor MG132 (20 μM) on p53 expression in NOP56-depleted HCT116 cells. **(D)** Effect of NOP56 depletion on the subcellular localization of p53 in cytoplasmic and nuclear fractions of HCT116 cells, as determined by Western blotting. **(E)** Effect of NOP56 depletion on the protein expression of p21, p27, Cyclin E, CDK4, CDK2, and MDM2 in HCT116 cells transfected with negative control or NOP56 siRNA. **(F)** Effect of NOP56 depletion on p53, p21, caspase-3 and PARP in HCT116 p53-/-, HepG2 (p53 WT), and Huh7 (p53 MT) cells transfected with negative control or NOP56 siRNA by Western blotting. WT, wild-type; MT, mutant.

Based on the NGS data, we further investigated whether NOP56 modulates the p53 signaling pathway. Western blot analysis revealed that NOP56 knockdown significantly increased p53 protein levels in p53 wild-type HCT116 cells, whereas no marked change was observed in p53-mutant SW620 cells (Figure 4A).

Because protein abundance reflects both synthesis and degradation rates 38, 42, we next examined p53 protein stability using cycloheximide (CHX) to inhibit *de novo* protein synthesis. In the presence of CHX, NOP56 depletion significantly prolonged the half-life of p53 compared with control cells, whereas MDM2 stability was reduced under the same conditions (Figure 4B). To determine whether NOP56 regulates p53 stability through the ubiquitin-proteasome pathway, HCT116 cells were treated with the proteasome inhibitor MG132. MG132 treatment alone increased p53 levels, and NOP56 silencing further enhanced MG132-induced p53 accumulation (Figure 4C).

To further assess the subcellular distribution of NOP56 and p53, nuclear and cytoplasmic fractionation was performed in HCT116 cells following siNOP56 transfection. NOP56 depletion markedly reduced its nuclear localization and was accompanied by a concomitant accumulation of p53 in the nucleus, suggesting that NOP56 negatively regulates nuclear p53 abundance (Figure 4D). Consistent with enhanced p53 stability and nuclear accumulation, depletion of NOP56 increased the expression of p53 downstream targets p21 and p27, while reducing Cyclin E, CDK4, CDK2, and MDM2 levels in HCT116 cells (Figure 4E).

To further validate p53 dependency, the effects of NOP56 depletion were compared in cell models with defined p53 status. NOP56 knockdown increased p53 and p21 expression and enhanced cleaved caspase-3 levels in p53 wild-type HepG2 cells, whereas these changes were not observed in HCT116 p53-/- or p53-mutant Huh7 cells (Figure 4F).

### NOP56 enhances p53 degradation by direct binding and colocalization in colorectal cancer cells

**Figure 5:**
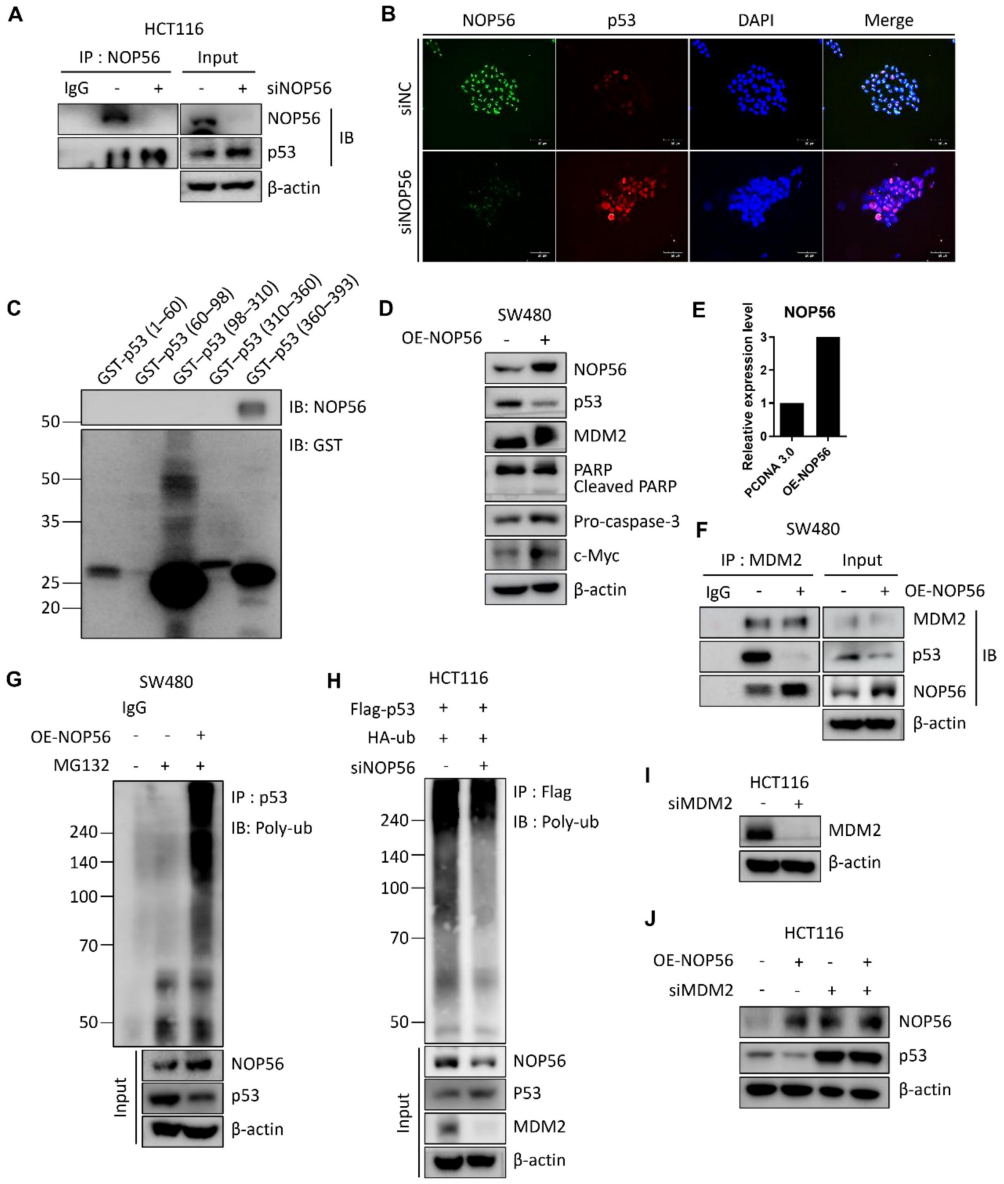
** NOP56 enhances p53 degradation through direct binding and colocalization in colorectal cancer cells. (A)** Co-immunoprecipitation analysis showing direct interaction between endogenous NOP56 and p53 in HCT116 cells. **(B)** Effect of NOP56 depletion on the nuclear colocalization of NOP56 and p53 in HCT116 cells, as determined by immunofluorescence staining. NOP56 (green), p53 (red), and DAPI (blue). Images were acquired at 100× magnification; scale bar, 50 μm. **(C)** GST pull-down assays were performed using GST-fused p53 domains corresponding to the transactivation domain (TAD, aa 1-60), proline-rich domain (PRD, aa 60-98), DNA-binding domain (DBD, aa 98-310), tetramerization domain (TD, aa 310-360), and C-terminal domain (CTD, aa 360-393). Bound proteins were analyzed by western blotting. **(D)** Effect of NOP56 overexpression on p53, MDM2, PARP, pro-caspase-3, and c-Myc protein expression in SW480 cells, as determined by Western blotting. **(E)** NOP56 mRNA expression in SW480 cells transfected with control or NOP56 overexpression plasmid, as analyzed by qRT-PCR. **(F)** Effect of NOP56 overexpression on the interaction between MDM2 and p53 in SW480 cells, as assessed by co-immunoprecipitation. **(G)** Effect of NOP56 overexpression on p53 ubiquitination in SW480 cells following MG132 treatment. **(H)** Effect of NOP56 depletion on p53 ubiquitination in HCT116 cells co-transfected with Flag-p53 and HA-ubiquitin. **(I)** Validation of MDM2 knockdown in HCT116 cells. **(J)** Effect of MDM2 depletion on p53 expression in HCT116 cells transfected with or without NOP56 overexpression (OE) plasmid.

Ubiquitin-proteasome degradation is a key mechanism regulating protein homeostasis in cancer cells 42. To investigate whether NOP56 directly binds to p53 in HCT116 cells, immunoprecipitation (IP), GST pull-down, and immunofluorescence analyses were performed. Co-immunoprecipitation confirmed an interaction between endogenous NOP56 and p53 (Figure 5A). Immunofluorescence analysis further demonstrated nuclear colocalization of NOP56 with p53, and NOP56 depletion resulted in reduced nuclear NOP56 signals accompanied by increased nuclear accumulation of p53 (Figure 5B). To determine whether the NOP56-p53 interaction is direct and to map the p53 region responsible for NOP56 binding, GST pull-down assay was performed using individual p53 domains, revealing that NOP56 specifically binds to the C-terminal domain (CTD) of p53 (Figure 5C).

To further assess the functional role of NOP56, NOP56 was ectopically expressed in SW480 cells, which exhibit low basal levels of mutant p53. Western blot analysis showed that NOP56 overexpression reduced p53 protein levels, while concomitantly increasing the expression of MDM2, c-Myc, and pro-caspase-3 (Figure 5D). qRT-PCR analysis confirmed successful overexpression of NOP56 at the mRNA level (Figure 5E).

Co-immunoprecipitation assays revealed that NOP56 overexpression enhanced the interaction between NOP56 and MDM2, while reducing the association between p53 and MDM2 (Figure 5F). Consistently, ubiquitination assays performed following MG132 treatment demonstrated that NOP56 overexpression promoted p53 polyubiquitination (Figure 5G).

To validate these findings in p53 wild-type cells, HCT116 cells were co-transfected with Flag-p53 and HA-ubiquitin in the presence or absence of NOP56 knockdown. NOP56 depletion markedly attenuated p53 ubiquitination, accompanied by increased p53 protein levels and reduced MDM2 expression (Figure 5H). To further determine whether NOP56-mediates p53 degradation and MDM2, MDM2 knockdown efficiency was validated in HCT116 cells (Figure 5I). Here NOP56 overexpression reduced p53 protein levels, leading to MDM2 depletion, indicating that NOP56-induced p53 degradation is dependent on functional MDM2 (Figure 5J).

Together, these findings indicate that NOP56 directly binds to the C-terminal domain of p53 and promotes its proteasomal degradation through MDM2-mediated ubiquitination, thereby attenuating p53 tumor suppressor activity in colorectal cancer cells.

### NOP56 regulates p53 acetylation at K382 by modulating SIRT1 and p300

**Figure 6:**
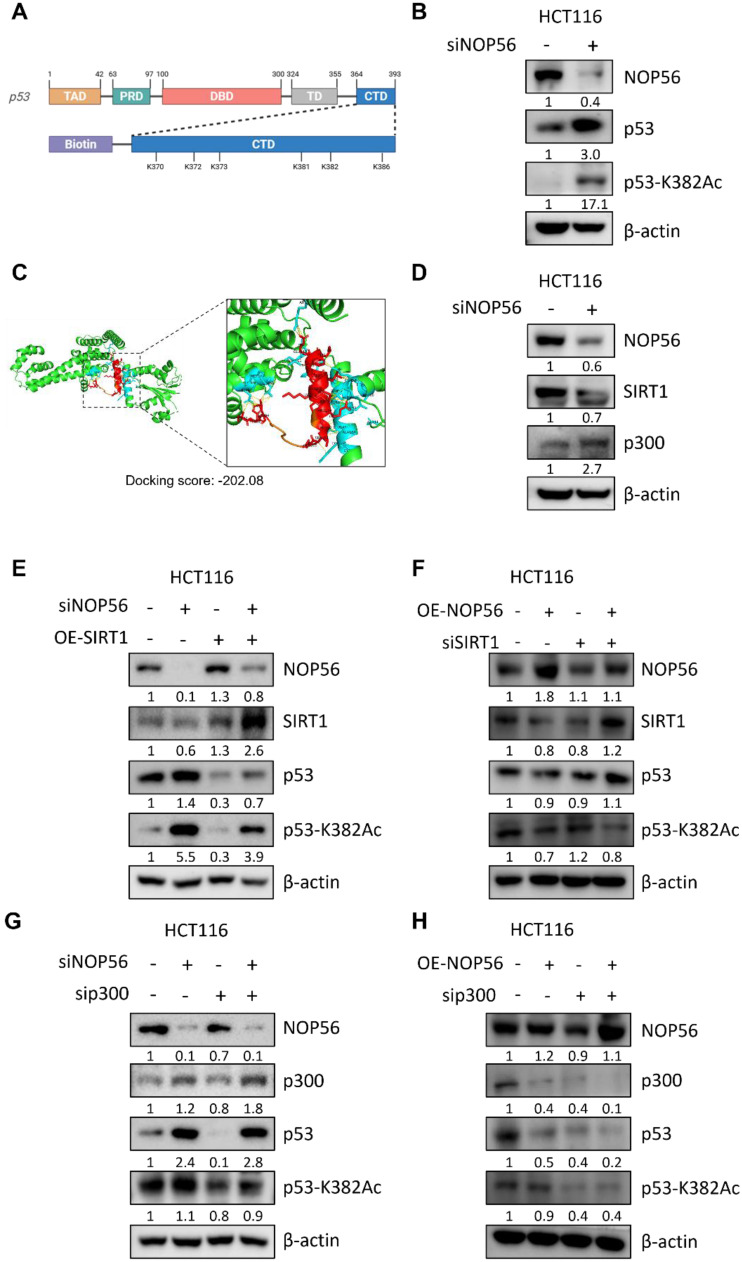
** NOP56 regulates p53 acetylation at K382 via the SIRT1/p300 signaling axis. (A)** Schematic representation of p53 domains, including the transactivation domain (TAD), proline-rich domain (PRD), DNA-binding domain (DBD), tetramerization domain (TD), and C-terminal domain (CTD). The CTD region is enlarged to highlight lysine residues (K370, K372, K373, K381, K382, and K386). **(B)** Effect of NOP56 depletion on total p53 and acetyl-p53 (K382) expression in HCT116 cells, as determined by Western blotting. **(C)** Structural models of the p53 DNA-binding domain (PDB: 1TUP) and the NOP56 N-terminal domain (PDB: 6ZDT). Docking analysis predicted a stable interaction between p53 and NOP56 (docking score: -202.08). **(D)** Effect of NOP56 depletion on SIRT1 and p300 protein expression in HCT116 cells, as analyzed by Western blotting. **(E)** Effect of SIRT1 overexpression on p53 acetylation in HCT116 cells in the presence or absence of NOP56 knockdown. **(F)** Effect of SIRT1 deletion on p53 acetylation in HCT116 cells transfected with or without NOP56 overexpression (OE) plasmid. **(G)** Effect of p300 depletion on p53 acetylation in HCT116 cells transfected with or without NOP56 depletion plasmid. **(H)** Effect of p300 deletion on p53 acetylation in HCT116 cells transfected with or without NOP56 overexpression (OE) plasmid.

We next investigated the role of NOP56 in p53 acetylation signaling. Acetylation of p53 is a reversible post-translational modification that regulates its transcriptional activity in response to cellular stress 43. Domain mapping of p53 revealed that multiple lysine residues, including K382, are clustered within the C-terminal regulatory domain (CTD) (Figure 6A). This region serves as a regulatory hotspot for ubiquitination, acetylation, and methylation, thereby modulating p53 stability, DNA-binding capacity, and transcriptional activity.

Western blot analysis showed that NOP56 knockdown in HCT116 cells increased both total p53 protein levels and acetylated p53 at K382 (Figure 6B), suggesting that NOP56 depletion enhances p53 stabilization through increased acetylation.

To further investigate potential structural interactions, we retrieved the crystal structures of the p53 DNA-binding domain (PDB: 1TUP) and the NOP56 N-terminal domain (PDB: 6ZDT) from the Protein Data Bank. Structural visualization revealed the relative orientation of p53 and NOP56, and docking analysis predicted a stable interaction, with a docking score of -202.08 and a confidence score of 0.739 (Figure 6C). The estimated binding free energy (ΔG) was -9.3 kcal·mol⁻¹, corresponding to a dissociation constant (Kd) of 1.6 × 10⁻⁷ M at 37 °C. Key interacting residues included LEU61-LEU108, GLY147-THR102, ARG154-HIS105, ARG154-MET109, VAL161-THR112, GLN170-LEU94, and ARG446-GLU113, with binding distances of 1.72-2.91 Å (Table 1). These findings indicate that NOP56 directly associates with the DNA-binding domain of p53 through multiple stabilizing contacts, providing a structural basis for NOP56-mediated regulation of p53 stability and its ubiquitination by MDM2. Consistent with increased p53 acetylation, knockdown of NOP56 reduced SIRT1 expression while increasing p300 levels in HCT116 cells (Figure 6D).

To further define the functional relationship between NOP56 and the SIRT1/p300 axis, genetic epistasis analyses were performed. Overexpression of SIRT1 reduced p53 acetylation, and this effect was attenuated by concomitant NOP56 knockdown (Figure 6E). Conversely, depletion of SIRT1 enhanced p53 acetylation, which was partially suppressed by NOP56 overexpression (Figure 6F). In parallel, depletion of p300 markedly reduced p53 acetylation, and this reduction was partially reversed by NOP56 knockdown (Figure 6G). In contrast, p300 depletion strongly suppressed p53 acetylation even by transfection with NOP56 overexpression plasmid (Figure 6H), indicating that p300 is a downstream of NOP56 for the regulation of p53 acetylation. Collectively, these results demonstrate that NOP56 regulates p53 acetylation at K382 through coordinated modulation of SIRT1 and p300, thereby contributing to functional inactivation of p53.

### NOP56 depletion sensitizes HCT116 cells to 5-fluorouracil (5-FU)

**Figure 7:**
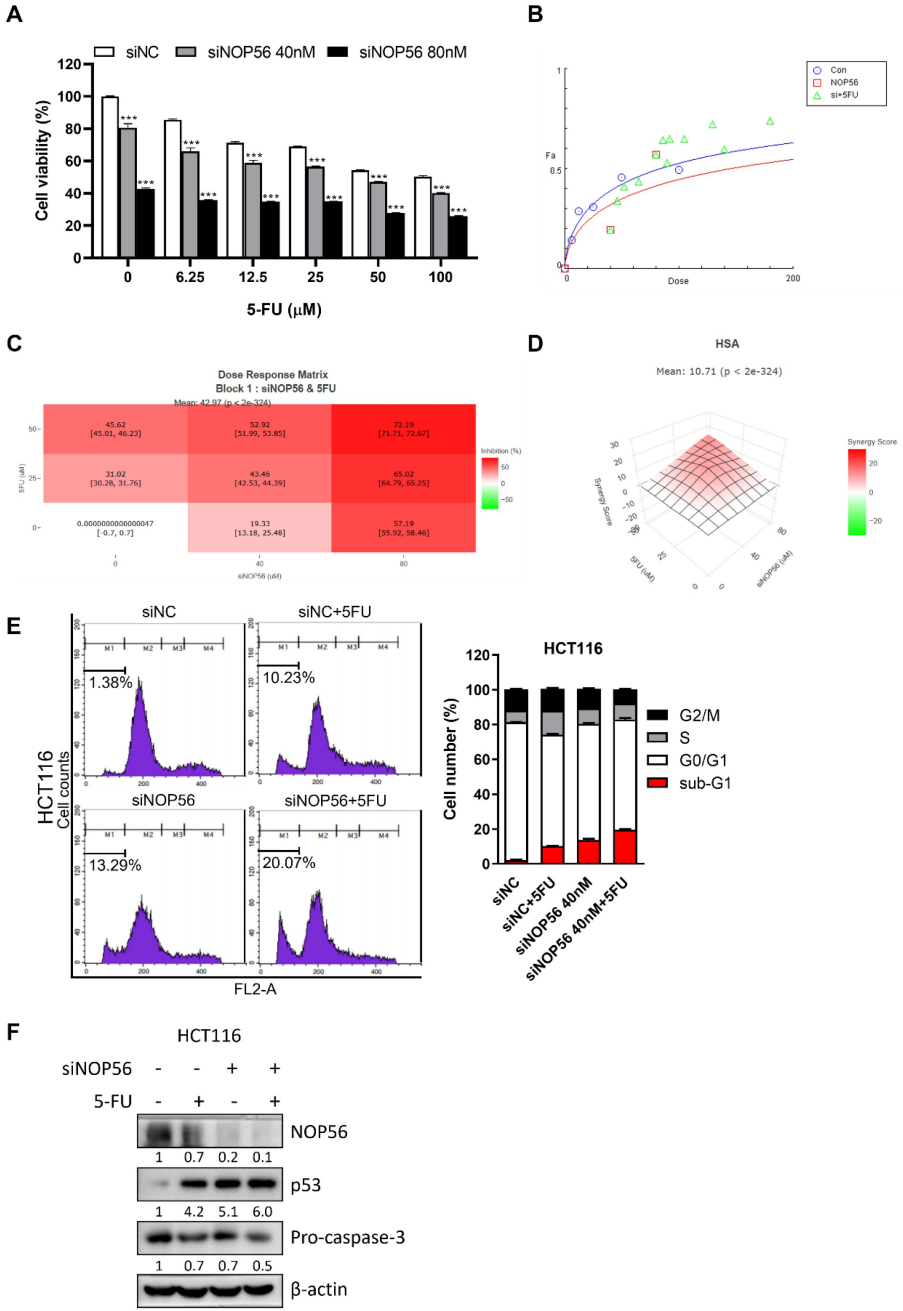
** Synergistic effects of NOP56 depletion and 5-FU in HCT116 cells. (A)** Effect of siNOP56 (0, 40, and 80 nM) and 5-FU (0-100 μM) on cell viability in HCT116 cells, as determined by MTT assay. ***p < 0.001 vs. 5-FU alone. **(B)** Dose-effect curve analysis of the combination of siNOP56 and 5-FU using CompuSyn software. Combination index (CI) values <1 indicate synergistic effects. **(C, D)** Synergy analysis of siNOP56 and 5-FU using SynergyFinder. Dose-response heatmaps show enhanced growth inhibition with the combination, and the highest single agent (HSA) synergy model indicates a mean synergy score of 10.71. **(E)** Effect of siNOP56 and 5-FU on cell cycle distribution in HCT116 cells, as analyzed by flow cytometry. Representative plots and quantification of the sub-G1 population are shown. **(F)** Effect of combined siNOP56 and 5-FU treatment on p53 and pro-caspase-3 protein expression in HCT116 cells, as determined by Western blotting. Data are presented as mean ± SD.

To determine whether NOP56 depletion enhances the chemosensitivity of colorectal cancer cells, MTT assays were performed in HCT116 cells treated with 5-fluorouracil (5-FU). NOP56 knockdown significantly potentiated 5-FU-induced cytotoxicity in a dose-dependent manner compared with treatment with 5-FU alone (Figure 7A).

Dose-effect analysis using CompuSyn software yielded combination index (CI) values of < 1, indicating synergistic interactions between NOP56 depletion and 5-FU (Figure 7B). Consistently, SynergyFinder analysis across selected dose ranges of siNOP56 (0, 40, and 80 nM) and 5-FU (0, 25, and 50 μM) demonstrated enhanced growth inhibition by the combination relative to either treatment alone, with a highest single agent (HSA) synergy score of 10.71 (Figure 7C, D).

Flow cytometric analysis further revealed that combined treatment with siNOP56 and 5-FU markedly increased the sub-G1 population (20.07%) compared with treatment with 5-FU (10.23%) or siNOP56 (13.29%) alone (Figure 7E). In parallel, Western blot analysis showed that the combination treatment further increased p53 protein levels and reduced pro-caspase-3 expression relative to either treatment alone (Figure 7F).

Collectively, these results demonstrate that NOP56 depletion synergistically enhances the cytotoxic and pro-apoptotic effects of 5-FU in p53 wild-type colorectal cancer cells.

### NOP56 depletion inhibits tumor growth of HCT116 cells in BALB/c nude mice

**Figure 8:**
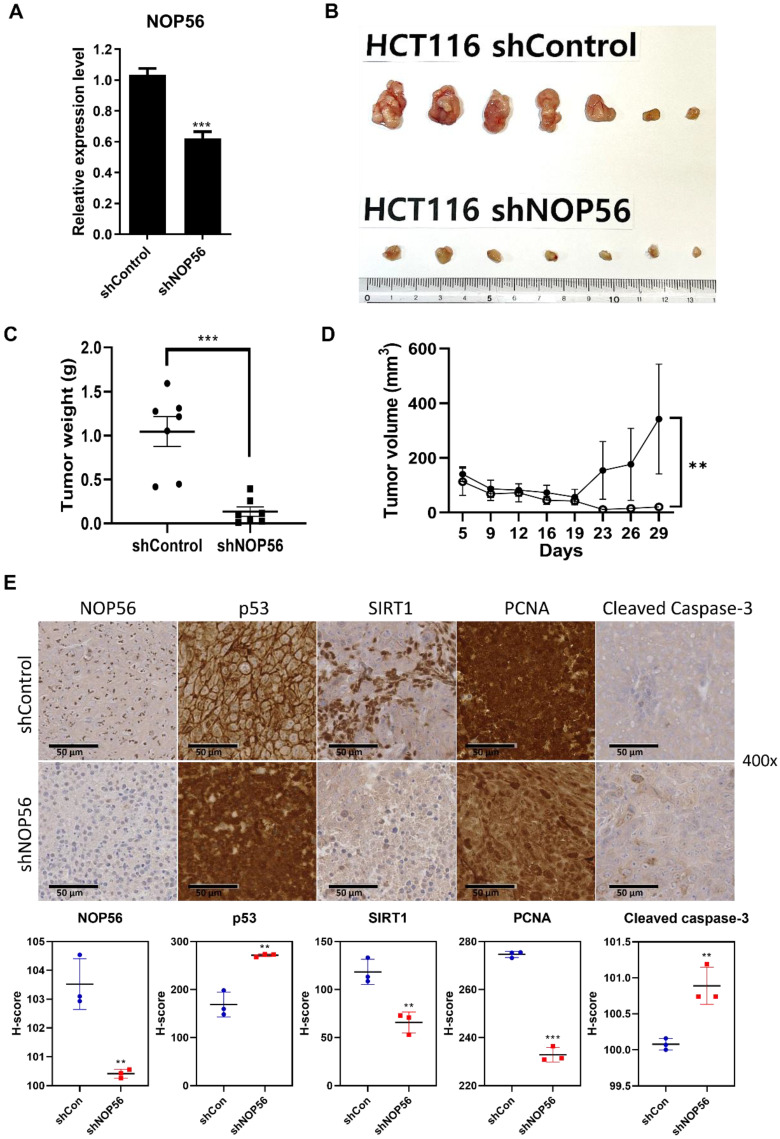
** NOP56 knockdown suppresses tumor growth of HCT116 xenografts in BALB/c athymic nude mice. (A)** Confirmation of NOP56 knockdown at the mRNA level in HCT116 cells transfected with control or NOP56 shRNA. **(B)** Representative images of tumors excised from mice implanted with shControl or shNOP56 HCT116 cells (n = 7 per group). **(C)** Tumor weights of xenografts from shControl and shNOP56 groups at the experimental endpoint. ***p < 0.001 vs. shControl. **(D)** Tumor volume growth curves of xenografts measured from 5 day to 28 day postimplantation . **p < 0.01 vs. shControl. **(E)** Immunohistochemical staining of NOP56, p53, SIRT1, PCNA, and cleaved caspase-3 in xenograft tumor tissues. Representative images are shown at 400× magnification.

To validate the *in vitro* findings, an *in vivo* xenograft model was established by subcutaneous injection of HCT116 cells stably transfected with control or NOP56 shRNA into male BALB/c athymic nude mice. Efficient knockdown of NOP56 was confirmed at the mRNA level in shNOP56-transfected HCT116 cells (Figure 8A). Functionally, NOP56 silencing markedly suppressed tumor growth *in vivo*. Representative images of excised tumors showed smaller xenografts in the shNOP56 group compared with controls (Figure 8B). Quantitative analysis revealed significantly reduced tumor volumes over a 35-day period, as well as decreased tumor weights at the endpoint, in the NOP56-depleted group (Figure 8C, D). Immunohistochemical analysis of xenograft tumor tissues further demonstrated reduced expression of NOP56, SIRT1, and the proliferation marker PCNA, accompanied by increased levels of p53 and cleaved caspase-3 in the shNOP56 group relative to controls (Figure 8E). Collectively, these results indicate that NOP56 depletion suppresses colorectal cancer tumor growth *in vivo* by enhancing p53 activation and apoptosis while attenuating proliferative signaling.

## Discussion

To develop advanced targeted therapies for colorectal cancer (CRC), we investigated the oncogenic mechanism of NOP56. NOP56, a core component of small nucleolar ribonucleoprotein complexes, has previously been reported to promote tumorigenesis through metabolic regulation and ROS homeostasis via the mTOR pathway in KRAS-mutant lung cancer cells 44. Nonetheless, its role in CRC has not been fully elucidated.

In the present study, TCGA analysis demonstrated that NOP56 is significantly overexpressed at the mRNA level in CRC tissues and is associated with poor survival. Elevated expression was further confirmed in CRC cell lines (HCT116, SW480, SW620, and HT-29), with particularly high levels in HCT116 and SW620. RNA sequencing (NGS)-based transcriptomic profiling of NOP56-depleted HCT116 cells revealed enrichment of genes related to DNA damage, apoptosis, and cell death, supporting an apoptotic role for NOP56 silencing.

Functionally, NOP56 depletion reduced viability, clonogenicity, and migration in HCT116 cells more strongly than in p53-mutant SW620 cells. Likewise, NOP56 knockdown increased p53 and p21 expression and enhanced cleaved caspase-3 levels in p53 wild-type HepG2 cells, not in p53 mutant Huh7 and p53 negative HCT116 p53-/- cells, highlighting p53 dependency. Consistent with this, NOP56 knockdown increased p53 and p21, while reducing c-Myc, Cyclin E, CDK2, CDK4, MDM2, and SIRT1. Conversely, NOP56 overexpression suppressed p53 and apoptotic markers, including PARP cleavage and caspase-3 activation, while activating MDM2. These results position NOP56 as a key regulator of p53 stability and apoptosis.

Mechanistically, NOP56 physically interacted and colocalized with p53, promoting MDM2-mediated ubiquitination and degradation of p53. Importantly, NOP56 depletion enhanced p53 acetylation at K382 by downregulating SIRT1 and upregulating p300, both of which are well-established regulators of p53 acetylation and transcriptional activity 21, 45, 46. Furthermore, functional rescue experiments demonstrated that SIRT1 overexpression suppressed, while p300 knockdown attenuated p53 acetylation and activity even in the context of NOP56 depletion, underscoring the central role of the SIRT1/p300 axis. In addition, genetic depletion of MDM2 abrogated the ability of NOP56 to reduce p53 protein levels, further supporting an MDM2-dependent mechanism of p53 degradation with SIRT1 silencing or p300 knockdown. These bidirectional manipulations consistently demonstrated that modulation of SIRT1 or p300 overrides the effects of NOP56 on p53 stability and acetylation**,** thereby establishing a hierarchical relationship in which NOP56 regulates p53 through the SIRT1/p300 axis.

Notably, the C-terminal domain (CTD) of p53, which we identified as the binding interface for NOP56, serves as a critical regulatory hub integrating multiple post-translational modifications, including ubiquitination and acetylation that govern p53 stability and transcriptional activity 26. Accordingly, the direct interaction between NOP56 and the p53 CTD provides a mechanistic framework for the coordinated regulation of p53 degradation and acetylation observed in this study.

Accumulating evidence indicates that c-Myc is a well-established oncogene that promotes tumorigenesis and progression in various cancers 47, 48. In our study, NOP56 depletion in HCT116 cells markedly reduced c-Myc expression while activating p53, as confirmed by qRT-PCR and Western blotting, thereby highlighting the pro-apoptotic effect of NOP56 silencing. *In vivo*, NOP56 knockdown significantly suppressed tumor growth in HCT116 xenografts, accompanied by increased p53 and cleaved caspase-3 expression and decreased NOP56, SIRT1, and PCNA levels.

In addition to its role as an oncogenic driver, our findings reveal that NOP56 depletion markedly sensitizes CRC cells to 5-FU, one of the most widely used chemotherapeutic agents for colorectal cancer. Synergy analyses using both CompuSyn and SynergyFinder consistently demonstrated that silencing NOP56 potentiates the cytotoxic effects of 5-FU in HCT116 cells, as evidenced by reduced cell viability, increased sub-G1 accumulation, and enhanced p53 activation and pro-caspase-3 cleavage. These results suggest that targeting NOP56 may enhance chemosensitivity to 5-FU by augmenting p53-dependent apoptotic signaling. However, although NOP56 suppression enhanced the cytotoxic effect of 5-FU *in vitro*, this observation is limited to cell-based assays. Thus, further *in vivo* validation will be required to substantiate the therapeutic potential of this combination in the future.

**Figure 9:**
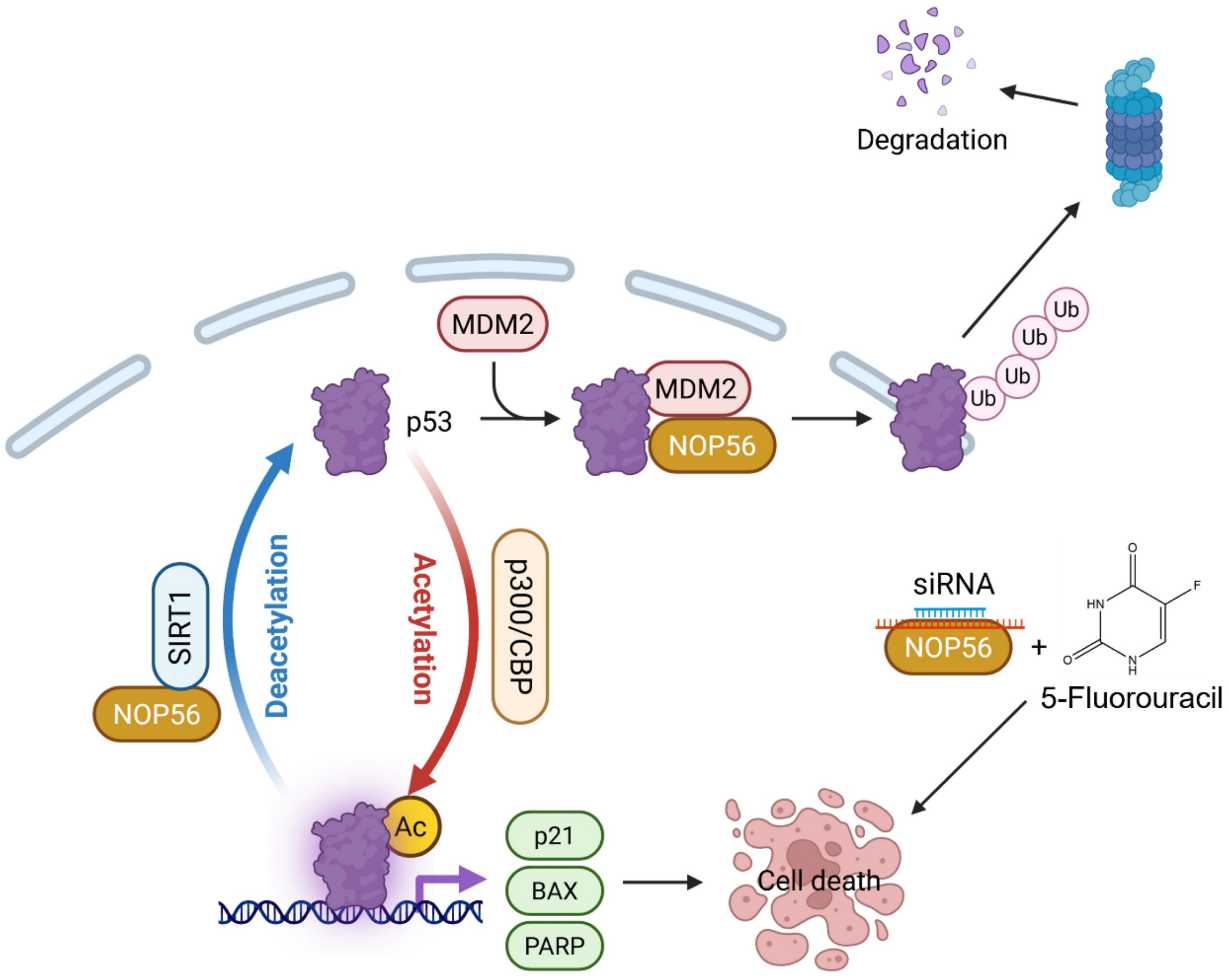
** Schematic representation of the apoptotic mechanism induced by NOP56 knockdown in colorectal cancer cells.** NOP56 depletion reduces MDM2-mediated p53 ubiquitination and alters SIRT1/p300-dependent p53 acetylation, leading to increased p53 stability and activity. Consequently, enhanced p53 signaling promotes apoptotic cell death and potentiates the cytotoxic effects of 5-fluorouracil (5-FU), highlighting a synergistic interaction between NOP56 silencing and chemotherapy.

Collectively, our findings identify NOP56 as an oncogenic driver in CRC, functioning to destabilize p53 and suppress its acetylation through regulation of SIRT1 and p300. Therapeutically, NOP56 inhibition restores p53 stability and acetylation, thereby inducing apoptosis and underscoring its potential as a promising target for the treatment of p53 wild-type CRC (Figure 9). Despite these promising results, this study has limitations. Combination experiments were limited to HCT116 (p53 wild-type) cells, warranting validation in p53-mutant or p53-deficient CRC models. *In vivo* xenografts confirmed tumor suppression by NOP56 knockdown; however, combination studies with 5-FU in animal models remain necessary. Future work should further assess transcriptomic and proteomic changes induced by dual targeting strategies and facilitate the development of small-molecule or RNA-based NOP56 inhibitors to enable clinical translation.

## Figures and Tables

**Table 1:** Docking score, binding energy, and interaction profile between NOP56 and p53.

Complex	Docking Score (kcal/mol)	Confidence Score	ΔG (kcal/mol-1)	Kd (M) at 37 °C	Receptor residue	Ligand residue	Distance (Å)
NOP56-p53	-202.08	0.739	-9.3	1.6 × 10⁻7	LEU61	LEU108	1.98
					GLY147	THR102	2.85
					ARG154	HIS105	2.91
					ARG154	MET109	2.75
					VAL161	THR112	2.87
					GLN170	LEU94	1.72
					ARG446	GLU113	2.34

## Data Availability

All experimental datasets from this study are available upon reasonable request from the corresponding author.

## Abbreviations

Bcl-2: B-cell lymphoma 2
Caspase: Cysteine aspartyl-specific protease
Caspase-3: cysteine-aspartic protease-3
CBP: CREB-binding protein
CNOT2: CCR4-NOT transcription complex subunit 2
CRC: colorectal cancer
CTD: C-terminal domain
DBD: DNA-binding domain
5-FU: 5-fluorouracil
FBS: Fetal bovine serum
MDM2: murine double minute 2
MTT: 3-(4,5-dimethylthiazol-2-yl)-2,5-diphenyltetrazolium bromide
NGS: next-generation sequencing
NOP56: nucleolar protein 56
OD: Optical density
PARP: poly(ADP-ribose) polymerase
PBS: Phosphate Buffered Saline
PRD: proline-rich domain
RIPA: Radio-immunoprecipitation assay
ROS: reactive oxygen species
RT-qPCR: real-time quantitative polymerase chain reaction
SDS-PAGE: Sodium dodecyl sulfate-polyacrylamide gel electrophoresis
TAD: transactivation domain
TBS-T: Tris-buffered saline with tween
TD: tetramerization domain
